# Looking into the life of technology-dependent children and their caregivers in Korea: lifting the burden of too many responsibilities

**DOI:** 10.1186/s12887-020-02388-z

**Published:** 2020-10-20

**Authors:** Yu Hyeon Choi, Min Sun Kim, Cho Hee Kim, In Gyu Song, June Dong Park, Dong In Suh, Hyung-Ik Shin

**Affiliations:** 1 Department of Pediatrics, Seoul National University Hospital, Seoul National University College of Medicine, 101 Daehak-ro, Jongno-gu, Seoul, 03080 South Korea; 2 Seoul National University College of Nursing, Seoul, South Korea; 3 Department of Pediatrics, Severance Children’s Hospital, Yonsei University College of Medicine, Seoul, South Korea; 4 Department of Rehabilitation Medicine, Seoul National University Hospital, Seoul National University College of Medicine, Seoul, South Korea

**Keywords:** Healthcare, Home care, Medical equipment, Home mechanical ventilation, Care burden, Cross-sectional study

## Abstract

**Background:**

The number of technology-dependent children (TDC) is increasing in South Korea, but available healthcare services after their discharge are poor. This study aimed to examine how TDC and caregivers live at home and identify their difficulties and needs regarding home care with few services to support them.

**Methods:**

This cross-sectional study was conducted in a tertiary hospital for children in South Korea. A self-reported questionnaire was completed by primary caregivers of TDC who were younger than 19 years and had been dependent on medical devices for more than 3 months. Technologies included home mechanical ventilation, oxygen supplementation, suction equipment, enteral feeding tube, and home total parenteral nutrition. Patterns of healthcare use and home care of TDC and caregivers’ perception toward child were assessed.

**Results:**

A total of 74 primary caregivers of TDC completed a self-reported questionnaire. About 60% children were aged under 5 years. There were 31.1% children who required both respiratory and nutritional support. On average, caregivers took care of a child for 14.4 (±6.1) hours, slept for 5.6 (±1.6) hours, and spent 2.4 h per day on personal activities. Children used hospital services for 41.3 (±45.6) days in 6 months, and most (78.1%) were transported through private car/ambulance. Participants (75.6%) reported taking more than an hour to get to the hospital. More than 80% of caregivers responded that child care is physically very burdensome. The only statistically significant relationships was between economic status and financial burden (*p* = 0.026).

**Conclusions:**

Caregivers of TDC reported having significant time pressure regarding childcare-related tasks, insufficient time for personal activities, and inefficient hospital use because of inadequate medical services to support them in South Korea. Thus, it is necessary to support caregivers and develop a home care model based on current medical environment.

**Supplementary information:**

The online version contains supplementary material available at 10.1186/s12887-020-02388-z.

## Background

With the advances in pediatric critical care and technology, number of children who require some form of technological assistance to compensate for impairment in vital function is increasing [1, 2]. These technology-dependent children (TDC) require an extensive range of healthcare services including preventive, specialty, emergent, and acute care services [1]. Recently, most TDC have maintained their medical care at home rather than in a hospital and this care usually goes on for a prolonged period after their acute treatments in inpatient facilities [3]. Hence, there is a growing demand for changes in healthcare services to improve care coordination with continuity, familiarity, accessibility, partnership, and early crisis recognition even at home [4]. In addition, it is suggested that someone responsible for these patients should be assigned to integrate community- and tertiary hospital-based services on behalf of the caregiver to ultimately relieve the caregivers’ burden.

Treatment outcomes of serious illnesses in children, such as survival in pediatric cancer, congenital heart disease, or prematurity in South Korea has significantly improved compared to that in other developed countries [5, 6]. As a result, the number of children who are temporarily or permanently dependent on technology is increasing. According to the national data in 2016, the prevalence of children with home mechanical ventilation (HMV) in South Korea is estimated at 4.4 per 100,000 for those aged under 19 years, which is similar to other countries [7]. In addition, it is suggested that the number of patients using HMV has increased by about three-fold from 2016 to 2018 [7]. Nevertheless, healthcare services for home care of TDC are poor in South Korea—there are no primary care physicians (PCPs) in the healthcare system [8], and no community- or home-based medical service for TDC and their families. Although there is an activity assistance service for the disabled, this service only provides non-medical support and is only available for those older than 6 years of age.

Thus, our hospital planned to develop a service for TDC after going back home, based on their needs as well as their caregiver’s. Until now, several studies have reported on the lives of TDC and their families, but most of these have been performed in areas where various healthcare services are already available. Therefore, we aimed to investigate the medical care environment at the homes of TDC with few healthcare service to support them, such as how to provide medical care at home, how to use hospital services, and what difficulties they have.

## Methods

### Setting, design, and participants

This cross-sectional study was conducted at the Seoul National University Children’s Hospital, which is a tertiary hospital for children, located in Seoul with 350 beds, including 24 designated beds for the pediatric intensive care unit. It is one of the few hospitals providing specialized medical service for pediatric patients in Korea; thus TDC are highly concentrated in this hospital regardless of where they live.

For eligibility, participants should have been primary caregivers of TDC. For this study, TDC was defined as patients younger than 19 years (0–18 years) with specialized medical equipment to support their lives, including HMV, oxygen supplementation, suction equipment, enteral feeding tube (nasogastric tube or gastrostomy tube, among others), or home parenteral nutrition [2]. The exclusion criteria were: not being a primary caregiver of TDC and if the care recipient was 19 years of age or older or had been dependent on medical devices for less than 3 months. The minimum duration of technology-dependency was adopted to ensure reasonable caregiving experience at home.

Before constructing a questionnaire, preliminary interviews with 12 primary caregivers of TDC were conducted to understand a primary caregiver’s experience regarding caregiving activities, intensity of care burdens, difficulties, and unmet needs. Once the questionnaire was developed, it was reviewed and revised by 15 multidisciplinary specialists: three pediatric intensivists, two neonatologists, three pediatric rehabilitation specialists, a pediatric pulmonologist, a gastroenterologist, a pediatric hematology/oncology specialist, two nurse unit managers, a clinical nutritionist, and a pharmacist. Lastly, the questionnaire was pilot tested and revised prior to being administered in this study.

The final version of the questionnaire (see Additional file 1) consisted of four sections: general characteristics (patients’ and caregivers’ age, sex, residence, type of insurance, financial status of family, educational level of caregiver, family types, and type and duration of technology-dependency; 13 items); the caregivers’ perception of their child’s medical condition and healthcare use (physical and psychological condition, physical ability, hospital admission, outpatient clinic visit with time spent, usage of emergency services, healthcare expenditure, and communication; 10 items); patterns of home care (detailed time spent on care, ways to get medical advice, caregiver burden, and difficulties; 21 items). The questions in this self-reported questionnaire were either categorical or used a 7-point Likert scale. In this questionnaire, health-related expenditures included hospital fee, fee for medication, medical equipment and supplies, transportation costs, nursing fee, rehabilitation, or any kind of therapy.

### Data collection

Data were collected from January 3 to February 14, 2019 using convenience and purposive sampling strategy. Trained researchers randomly approached and screened the eligibility of caregivers while they are waiting for their child’s outpatient appointments. After providing information about the study and obtaining their written informed consent, a total of 74 caregivers participated and completed the questionnaire. They took approximately 20 min on average to respond to the whole questionnaire.

### Statistical analysis

Data were analyzed using descriptive statistics and presented through mean and standard deviations, while frequencies and percentages were used in case of categorical variables. We used univariate binary logistic regressions to examine the association of care burden with demographic (child’s age, child’s sex, residential area, child’s insurance type, family types, caregiver’s perceived financial status of the family, and caregiver’s level of education) and clinical (primary diagnosis, physical capacity, level and duration of technology-dependency) variables. Those that were statistically significant in univariate analysis were then included in the multiple logistic regression to examine their associations with care burden. Care burden or difficulties were dichotomized by combining responses of 5, 6, and 7 (Very much) into the considerable category, and others into a not considerable category, using the median score to differentiate “considerable” and “not considerable” [9]. All analyses were performed using IBM SPSS Statistics version 20 (IBM Company, Chicago, IL, USA), and the level of significance was defined as *p* < 0.05.

## Results

### General and clinical characteristics

A total of 74 TDC and their respective caregivers were included in the study (Table 1). The mean age of the children was 5.7 (± 5.2) years, with 46 patients (62.2%) aged 5 years and below. Fifty patients (67.6%) lived in Seoul and its surrounding cities (Incheon and Gyeonggi). Airway and respiratory diseases were the most common primary diagnosis, followed by neurological diseases. There were 16 (21.6%) and 24 (32.4%) patients who could walk and sit without help, respectively. About 70% of patients had been receiving technology aid for more than 1 year. Mothers were the most common primary caregivers (89.2%).

**Table 1:** General and clinical characteristics of technological-dependent children and their caregivers (*n* = 74)

Variables	Categories	No	(%)
Child’s age (in years)	< 1	5	6.8
	1–5	41	55.4
	6–11	17	23.0
	12–18	11	14.9
Child’s sex	Male	53	71.6
	Female	21	28.4
Area of residence	Seoul	21	28.4
	Incheon, Gyeonggi	29	39.2
	Others	24	32.4
Child’s insurance type	National health insurance	64	86.5
	Medical aid program	10	13.5
Family’s financial status	High	9	12.2
	Middle	27	36.5
	Low	38	51.4
Primary caregiver	Mother	66	89.2
	Father	2	2.7
	Grandparent	2	2.7
	Others^a^	4	5.4
Caregivers’ educational level	High school level or lower	19	25.7
	Bachelor’s degree or higher	55	74.3
Family types^b^	Two-parents	67	90.5
	Single-parent	6	8.1
Primary diagnosis	Cardiovascular	4	5.4
	Airway/respiratory disease	21	28.4
	Neuromuscular disease	15	20.3
	Neurologic disease	19	25.7
	Others	15	20.3
Physical capacity^c^	Able to walk	16	21.6
	Able to stand briefly	23	31.1
	Able to sit independently	24	32.4
	Able to sit with help	30	40.5
	Able to roll over	36	48.6
	Able to lift head	42	56.8
	None (N/A)	32	43.2
Duration of technology-dependency	< 1 year	21	28.4
	1–3 years	22	29.7
	≥ 3 years	31	41.9

^a “^Others” included an activity assistant service for persons with disabilities, a neighbor, a day care center for persons with disabilities, and a teacher at a special school

^b^ One caregiver did not respond

^c^ Five children were under 1 years old and could neither walk nor stand independently

As shown in Table 2, all TDC used more than one medical device. Regarding type of respiratory support device, a tracheostomy tube and a HMV were used by 42 (56.8%) and 36 (48.7%) patients, respectively. A total of 36 patients (48.6%) required a nasogastric tube or a gastrostomy tube, and five needed an intravenous pump for parenteral nutrition. Additionally, the number of children requiring both respiratory and nutritional devices was 41 (55.4%).

**Table 2:** Type of support device for technology-dependent children

	Respiratory support									
Nutrition support	O2 only^a^		Tracheostomy without ventilator		Non-Invasive Ventilator		Tracheostomy and ventilator		Total	
Oral feeding, *n* (%)	5	(6.8)	12	(16.2)	7	(9.5)	9	(12.2)	33	(44.6)
Enteral feeding, *n* (%)	13	(17.6)	3	(4.1)	2	(2.7)	18	(24.3)	36	(48.6)
Parenteral feeding, *n* (%)	5	(6.8)	0	(0.0)	0	(0.0)	0	(0.0)	5	(6.8)
Total, *n* (%)	23	(31.1)	15	(20.3)	9	(12.2)	27	(36.5)	74	(100.0)

^a^ included patients who received non-invasively oxygen without tracheostomy or home mechanical ventilation

### Patterns of medical care at home

**Fig. 1:**
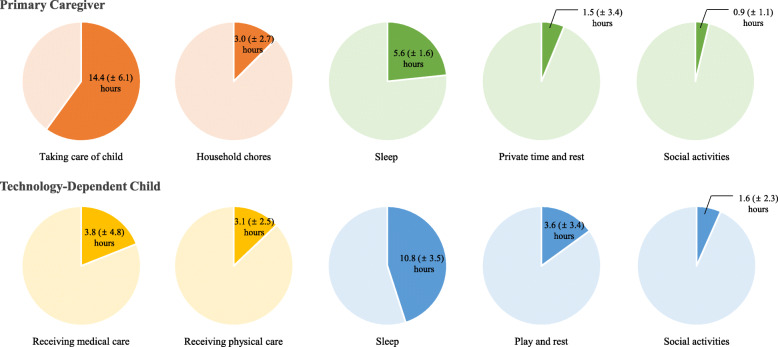
Daily activity patterns for technology-dependent children and their caregivers. Data are presented as “mean ± standard deviation”. Each circle means 24 h a day. Examples of each activity are as follows: taking care of child includes any activity provided by caregivers to their technology-dependent child (supervision as well as medical and physical care, etc); household chores (vacuuming, doing the laundry, taking care of other family members, etc.); private time and rest (watching TV, surfing the internet, playing games, reading, etc.); social activities (going to school, meeting friends, working, etc.); receiving medical care (taking medicine, suctioning, changing tracheostomy tube, etc.); receiving physical care (bathing, eating, dressing, etc.)

The TDC slept an average of 10.8 (± 3.5) hours and received medical or physical care for 6.9 h each day (Fig. 1). Caregivers spent most of their time on childcare-related tasks, including medical care for 14.4 (± 6.1) hours a day. With regard to the remaining time, 5.6 (± 1.6) hours were spent on sleep, and only 2.4 h per day were spent on personal activities, such as resting and hobbies.

The specific daily childcare-related tasks by the caregivers and their corresponding frequency per day are as follows: suctioning (17.7 times/day), position change (6.8 times/day), feeding via nasogastric or gastrostomy tube (6.4 times/day), rehabilitation (3.8 times/day), disinfection or cleaning of medical instrument (2.4 times/day), and administration of medication (1.8 times/day).

### Patterns of healthcare utilization

TDC used hospital services for an average of 41.3 (± 45.6) days in the last 6 months (Table 3). About 80% used a private car or a privately paid ambulance to access the hospital. Among the participants, 75.6% reported that it took more than an hour to get to the hospital and nearly half of all patients had to wait in the hospital for more than an hour again to consult with the physician. The actual consultation time was less than 10 min in 82.4% of cases, and only 4.1% said they spent over 15 min with a physician. Additionally, the order of preference for the way in which caregivers got medical advice was as follows: ask acquaintances or self-help group, 76.4%; contact hospital or medical staff, 59.7%; and search internet, 50.0%. Even when contacting the hospital, 36.5% had to wait until their outpatient appointment.

**Table 3:** Patterns of healthcare services utilization for technology-dependent children and their caregivers

Variables	Responses	n (%) or mean ± *SD*
Healthcare use within recent 6 months (days) ^a^	All hospital days combined	41.3 ± 45.6
	Number of days visiting OPD	18.2 ± 30.0
	Length of stay in the general ward	17.0 ± 30.3
	Length of stay in the intensive care unit	5.1 ± 20.6
	Number of days visiting ED	1.5 ± 1.5
Transportation^b^	Private car or private ambulance	57 (78.1)
	Public transport (bus, subway, train, etc.)	14 (19.2)
	Public transportation support	10 (13.7)
Travel time (from home to hospital)	< 1 h	18 (24.3)
	≥1 h, < 3 h	36 (48.6)
	≥3 h	20 (27.0)
Outpatient waiting time in the hospital	< 30 min	12 (16.2)
	≥30 min, < 1 h	27 (36.5)
	≥1 h, < 2 h	23 (31.1)
	≥2 h	12 (16.2)
Outpatient consultation time in the hospital (physician encounter time)	< 5 min	29 (39.2)
	≥ 5 min, < 10 min	32 (43.2)
	≥10 min, < 15 min	10 (13.5)
	≥15 min	3 (4.1)
Rehabilitation (within recent 3 months)	Both (at hospital and home)	22 (29.7)
	At hospital	19 (25.7)
	At home	4 (5.4)
	None	29 (39.2)
How to get medical advice^b^	Ask acquaintance or self-help group	55 (76.4)
	Contact hospital or medical staff	43 (59.7)
	Search Internet	36 (50.0)
	Search professional books	5 (6.9)
Ways to contact the hospital^b^ (when the caregiver needs medical advice at home, within the last year)	Wait until planned OPD visit or admission	27 (36.5)
	Never have asked to any hospital related staff	26 (35.1)
	Call to the ward where child was previously hospitalized	22 (29.7)
	Call to a pediatric ED	7 (9.5)
	Call to a palliative care team (if applicable)	6 (8.1)
	Others ^c^	3 (4.1)

*SD* standard deviation, *OPD* outpatient department, *ED* emergency department

^a^ Of the 74 participants, seven responses were excluded, leaving a total of 67 responses for healthcare use analysis. Reasons for exclusion included 2 missing values and 5 responses exceeding 180-day within 6 months

^b^ There were multiple responses

^c^ “Others” included someone who is a doctor, a pharmacist, or a physical therapist

### Care burden and difficulties perceived by caregivers

The vast majority (82.4%) of caregivers in this study reported physical burden, followed by psychological and financial burdens (Table 4). Regarding specific difficulties, 87.5% of caregivers reported that they had no one to take care of the child while resting and 86.1% said they needed someone’s help to take care of the sick child. Caregivers also mentioned that they lack the energy and time for household chores or to take care of other family members. More than three-fourths of participants indicated traveling to the hospital and having to pay for medical expenses to care for the child as reasons for their difficulties. In addition, caregivers said that they did not know how to take care of TDC or that they needed help with the procedures at home.

**Table 4:** Levels of care burden and specific difficulties perceived by caregivers

Variables	Mean ± *SD*	*n*^c^	(%)
**Perceived care burden**^a^			
Physical burden	5.5 ± 1.4	61	(82.4)
Psychosocial burden	5.1 ± 1.5	51	(68.9)
Financial burden	4.9 ± 1.5	43	(58.1)
**Specific difficulties**^b^			
No manpower to care for the child while the caregiver is resting	6.0 ± 1.4	63	(87.5)
Need someone’s help to take care of the sick child	6.0 ± 1.4	62	(86.1)
Lack of energy and time to do household chores	6.9 ± 1.4	62	(86.1)
Always tired	5.9 ± 1.4	61	(84.7)
Lack of energy and time to care for another child or family	5.6 ± 1.8	57	(79.2)
Lack of support when travelling to hospital	5.3 ± 2.0	56	(77.8)
Substantial amount of medical expenses	5.5 ± 1.8	50	(69.4)
Lack of knowledge on how to take care of the sick child	4.9 ± 1.8	44	(61.1)
Need help with some medical procedures at home	4.6 ± 2.3	44	(61.1)
Hard to coordinate hospital appointments	4.6 ± 2.2	42	(58.3)
Had to forgo some medical care due to financial reasons	3.9 ± 2.4	31	(43.1)
Need help from healthcare professionals for care at home	3.7 ± 2.2	28	(38.9)
Lack of knowledge on how to manage medical devices	3.6 ± 2.0	26	(36.1)

*SD* standard deviation

^a^Perceived level of care burden and difficulties were measured on a 7-point Likert scale ranging from 1 (*not at all*) to 7 (*very much*)

^b^The list below was derived from interviews conducted before constructing the questionnaire

^c^Burden and difficulties were dichotomized by combining responses of 5, 6, and 7 (*very much*) into considerable (burden or difficulty) category, and others into a not considerable category

### Factors affecting the level of perceived care burden

There were no statistically significant relationships between any variable, including child’s age, residential area, financial status, level of education, family types, level of technology dependence, and level of perceived burden, except that between economic status and economic burden in multivariate analysis (OR 7.414, CI 1.275–43.116, *p* = 0.026) (Table 5).

**Table 5:** Factors affecting level of perceived care burden (*n* = 74)

Variables	Physical burden		Psychosocial burden		Financial burden	
	OR (95% CI)	*p* value	OR (95% CI)	*p* value	OR (95% CI)	*p* value
**Child’s age (years)**						
< 1	Reference		Reference		Reference	
1–5	1.031 (0.101–10.53)	0.979	1.818 (0.267–12.376)	0.541	0.238 (0.024–2.317)	0.216
6–11	1.167 (0.094–14.518)	0.905	0.952 (0.125–7.275)	0.962	0.458 (0.041–5.085)	0.525
12–18	2.5 (0.124–50.444)	0.550	1.778 (0.192–16.492)	0.613	0.667 (0.051–8.639)	0.756
**Child’s sex**						
Male	Reference		Reference		Reference	
Female	0.569 (0.162–1.995)	0.378	0.641 (0.221–1.86)	0.414	0.722 (0.261–1.998)	0.53
**Area of residence**						
Seoul (capital city)	Reference		Reference		Reference	
Incheon, Gyeonggi	1.042 (0.207–5.237)	0.960	1.969 (0.6–6.455)	0.264	1.8 (0.577–5.619)	0.312
Others	0.5 (0.108–2.314)	0.375	2.25 (0.635–7.973)	0.209	1.833 (0.558–6.027)	0.318
**Insurance type**						
National Health Insurance	Reference		Reference		Reference	
Medical aid	2.077 (0.240–17.995)	0.507	1.953 (0.381–10.02)	0.422	3.314 (0.652–16.844)	0.149
**Financial status**						
High	Reference		Reference		Reference	
Medium	0.816 (0.136–4.898)	0.824	1.9 (0.402–8.976)	0.418	3.25 (0.569–18.579)	0.185
Low	2.429 (0.370–15.951)	0.356	1.964 (0.443–8.713)	0.375	9.8 (1.738–55.246)	**0.010** ^c^
**Level of education, caregiver**						
High school level or lower	Reference		Reference		Reference	
Bachelor’s degree or higher	0.844 (0.206–3.458)	0.813	1.032 (0.335–3.174)	0.957	0.277 (0.081–0.940)	0.039
**Family types**						
Two-parents	Reference		Reference		Reference	
Single-parent	0.982 (0.104–9.246)	0.987	0.851 (0.144–5.028)	0.859	3.816 (0.422–34.463)	0.233
**Primary diagnosis**						
Cardiovascular	Reference		Reference		Reference	
Airway/respiratory disease	2.00 (0.153–26.187)	0.597	0.833 (0.072–9.688)	0.884	1.1 (0.13–9.339)	0.930
Neuromuscular disease	4.667 (0.223–97.497)	0.321	2.167 (0.144–32.528)	0.576	4.0 (0.388–41.228)	0.244
Neurologic disease	1.25 (0.101–15.499)	0.862	0.370 (0.032–4.231)	0.424	1.111 (0.129–9.605)	0.924
Others	0.917 (0.173–11.577)	0.946	0.667 (0.054–8.161)	0.751	1.143 (0.126–10.386)	0.906
**Physical capacity**						
Good^a^	Reference		Reference		Reference	
Poor	3.02 (0.886–10.286)	0.077	1.543 (0.55–4.328)	0.410	1.632 (0.610–4.360)	0.329
**Level of technology-dependency**						
Low dependency	Reference		Reference		Reference	
High dependency^b^	1.288 (0.316–5.256)	0.724	0.577 (0.197–1.69)	0.316	0.481 (0.17–1.361)	0.168
**Duration of technology-dependency**						
Less than 1 year	Reference		Reference		Reference	
1–3 years	1.406 (0.321–6.160)	0.651	1.641 (0.453–5.943)	0.451	1.32 (0.398–4.378)	0.65
3 years or more	2.109 (0.493–9.019)	0.314	1.504 (0.465–4.864)	0.495	2.31 (0.739–7.226)	0.15

*OR* odds ratio, *CI* confidence interval

^a^Good physical capacity includes patients who are able to walk, stand briefly or sit independently

^b^ High technology-dependency indicates patients with both enteral nutrition support (ex. nasogastric tube, gastrostomy tube, etc.) and respiratory support (tracheostomy and/or home mechanical ventilator)

^c^The only statistically significant variable in multivariate logistic regression analysis. (OR 7.414, CI 1.275–43.116, *p* = 0.026)

## Discussion

In this study, the majority of the caregivers described their experiences of spending a considerable time on childcare, with insufficient private time and frequent long-distance hospital usage with short consultation time in the outpatient clinic. Thus, the caregivers suffered from various physical, psychological, and financial burdens. We focused on TDC, who have more complex medical conditions, and found that caregivers’ burdens were not related to the severity of the child’s condition. To the best of our knowledge, this is the first study that specifically quantified the childcare-related tasks of TDC caregivers, daily activity patterns of both the child and the caregiver, and TDC’s utilization patterns of the healthcare system, such as transportation, with few community- or hospital-based medical home services to support them.

Caregivers of children with medical complexities perform a variety of activities that are usually conducted by different professionals in a hospital, such as those performed by nurses and consulting physicians, as well as parenting for a limited time [10]. McCann et al. reported that caregivers spend approximately 63 to 726 min per day doing all these care activities [11]. In this study, with a narrower population of TDC, parents spent more than half of the day on childcare-related tasks, which led to absolute lack of personal time. Additionally, given the number of specific daily care-related tasks presented in this study, we estimated that one or more essential tasks were performed every hour. Consequently, activities other than caring of children, including sleep, are expected to be usually fragmented and scattered without continuity throughout the day. As reported by previous studies, these activities eventually affect family functioning or reduce social activities, resulting in the caregivers’ social isolation [3, 12].

TDC utilize a tertiary hospital service, including outpatient clinics, intensive care unit, and emergency departments, with a high frequency [13, 14]. The results of this study are consistent with that of previous reports; however, we found that our participants used hospitals more inefficiently. Many children in this study lived in areas that are more than an hour away from the hospital by car. Moreover, they had to come to an outpatient clinic at least once a month to see a doctor for less than 10 min on average. We believe that this inefficiency in hospital use led to the several consequences reported in this study. First, when health related problems occur during medical care at home, caregivers usually seek medical advice mainly from non-medical sources such as other caregivers and the internet. In case of seeking advice from medical practitioners, they were more likely to wait for a scheduled appointment rather than immediately contact a medical staff. This can cause management errors related to the treatment of TDC that prevent them from receiving it in a timely and adequate manner [15]. Second, TDC repeat long-distance travels with essential medical devices, which can eventually exhaust caregivers and add more negative outcomes related to care burden [16]. Third, considering that the primary means of transportation was a private car or an ambulance, which are not covered under the national insurance in Korea, it is possible that frequent hospital visits are burdens on household finances.

Among the several obstacles for parents of children with serious illnesses, physical exhaustion has emerged as the major hurdle in our study. Notably, the top 5 specific difficulties described by caregivers were also associated with physical burdens. Interestingly, our results showed no statistically significant relationship between caregivers’ burdens and the medical conditions or general characteristics of children. However, one Japanese study involving children with special healthcare needs, including TDC, showed that tracheostomy with home ventilators for older groups of children and older siblings for younger groups of children increased caregiver burden [17]. It is presumed that this inconsistent result is owing to the difference in patients’ characteristics. TDC, included in this study, presented a higher medical complexity and lower functional status, with more than half below 5 years; hence, they had more needs that caregivers need to attend to [11]. Thus, since the burdens of TDC caregivers are already considerable, they might be significantly more affected by their caring environment rather than by demographic or medical conditions. This result support that home care environment with lack of healthcare system to support TDC leads to higher intensity parents-led care, which ultimately increase caregiver’s burdens.

Therefore, appropriate services should be developed to relieve caregivers’ burdens at home and deliver continuous and coordinated medical services for TDC. The American Academy of Pediatrics recommends that the PCPs of a community should be matched with children during the transition period between care at the hospital and care at home [18]; however, there are no PCPs in the South Korea healthcare system [8]. Furthermore, tertiary hospitals are responsible not only for specialist care related to diseases among TDC, but also to their routine, emergency, and critical care. Thus, compared to most developed countries where the primary physician system of a community is well established, other countries, where such a system is insufficient, need an alternative.

Recently, hospital-based comprehensive care program are steadily increasing in children with complex conditions who are closely connected with tertiary care centers [4, 19]. This model can be particularly helpful for TDC who frequently require discussions with multiple subspecialties owing to their complex issues. As a result, through reorganizing the current care system that is centered on a tertiary hospital, it is expected to produce positive outcomes, such as efficient hospital usage and appropriate medical care at home. However, this care model requires substantial financial, structural, and staff resources in a tertiary hospital [4]. Additionally, because multiple teams are involved, there is a risk of diffusion of responsibility, given the ambiguous patient ownership and lack of integration with community-based services. In a recent study, the accessibility of services were improved and caregiver’s satisfaction were increased through the designated care coordinator in a tertiary hospital, such as complex care team [20]. Nevertheless, in our study, since caregivers mainly experienced daily absolute “overwork” associated with caring for their child, enrollment in a certain model of care for TDC could be insufficient to relieve parents’ burdens or improve their quality of life [21]. Thus, additional systems that free them from “home,” such as respite facilities or programs, should be considered. Consequently, depending on the resources in a hospital and community, it is ultimately necessary to modify the services in accordance with the perspective of the caregivers in detail, and not the medical staff, as has been done in our study.

This study has some limitations. First, it was conducted in a single institution and had a small sample, which limits the representation and generalization of the findings. However, since the chosen research institute is, currently, one of the major tertiary hospitals located in the capital of South Korea, it can be said that the findings offer an average understanding of the specificities of TDC’s care environment. Nevertheless, we believe that future studies need to work with bigger samples and include more institutions. Second, this study used a cross-sectional design with a self-reported questionnaire that was completed by the TDC’s main caregivers; thus, recall bias may have affected their responses, and consequently, our findings. Third, because of the limitations of using a self-reported questionnaire, we could not collect information regarding co-morbidities of TDC. Thus, unlike previous studies, the accompanying diseases, except for the main diagnoses, could be underestimated in this study. Fourth, because participants were selected from outpatients, as per the inclusion criteria, selection bias could have also impaired our results. We believe that future studies must provide other types of reports for questionnaires, and sample selection should be randomized to allow generalization of the findings.

## Conclusions

Lack of home care support causes significant burdens for TDC caregivers as well as inefficient hospital usage and occasional inappropriate medical care at home. Our study suggests that TDC require comprehensive care services, which should be modified based on the limited resources and needs of caregivers.

## Supplementary information

Additional file 1. Questionnaire in English. (PDF 254 kb)

## Data Availability

Data supporting the results are available from the corresponding author upon request.

## Abbreviations

HMV: Home mechanical ventilation
PCPs: Primary care physicians
TDC: Technology-dependent children
