# Supplementary material for: Looking into the life of technology-dependent children and their caregivers in Korea: lifting the burden of too many responsibilities
Source: BMC Pediatr. 2020 Oct 20;20:486. doi: 10.1186/s12887-020-02388-z (PMC7572933; doi:10.1186/s12887-020-02388-z)
Supplement: Supplementary file 1 — Questionnaire in English. (PDF 254 kb) [file 12887_2020_2388_MOESM1_ESM.pdf]

A survey on Home Care for Critically Ill Children and Adolescents

SQ1) Are you a mjor caregiver for the pediatric patient?

1. Yes　　　2. No (--> Stop survey)

SQ2) How old are the pediatric patient?

_________ years __________ months (--> Stop survey if more than 19 years old)

SQ3) What is the gender of the pediatric patient?

1. Male　　　2. Female

SQ4) Does the pediatric patient always use the following equipment because of the disease? Please select all the equipment the pediatric patient use, all the time.

1. Tracheostomy tube
2. Home mechanical ventilation
3. Home parenteral nutrition
4. Nasogastric tube, gastrostomy tube
5. Pulse oxygen saturation monitor
6. Home oxygen concentrator
7. Suction equipment
8. Other (Please specify;__________________________)

SQ5) How long has the pediatric patient been using such a equipment? (Based on the time of first use)

1. Less than 3 month
2. 3 ~ 6 month
3. 6 month ~ 1year
4. 1 ~ 2 year
5. 2 ~ 3 year
6. More than 3 year

(--> Stop survey if the answer is '1')

I. Questions on the condition and treatment of pediatric patients.

Q1) What is your child's primary diagnosis?

____

Q2) What is the current **severity of the pediatric patient's illness**?
Please select between 'A great deal (Very problematic)'(7) and 'Not at all (Not a problem)'(1) based on what you know about the severity of the pediatric patient's illness.

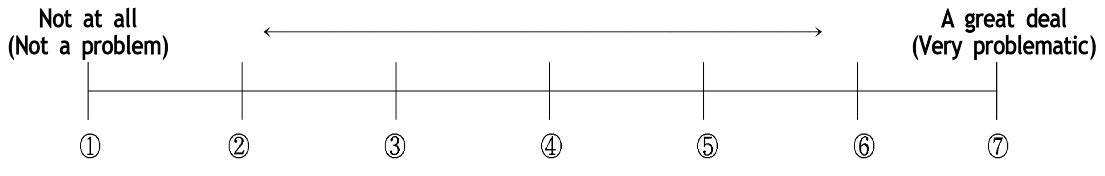

Q3) How is the current **communicative ability of the pediatric patient**? Please select the answer that best describes it.

Copyright(c) 2019 Consensus Co. All rights reserved.

①	②	③
Able to cry, smile, make sounds, and use body language to express themselves but unable to engage in deliberate communication	Cry, smile, make sounds, and use body language to engage in deliberate communication with a purpose	Use words, pictures, and sign language to engage in clear communication and exchange intentions

Q4) How is the current **physical ability of the pediatric patient**? Please select **all** that the **pediatric patient can do alone**.

①	②	③	④	⑤	⑥	⑨
Walk	Stand briefly	Sit independently	Sit with help	Roll over	Lift head	Unable to do none of the above

Q5) How do you assess the current **degree of physical and psychological difficulties** faced by the pediatric patient? Please select between 'A great deal (Very problematic)'(7) and 'Not at all (Not a problem)'(1) based on your assessment of the condition of the pediatric patient.

Variables	Not at all (Not a problem) ... A great deal (Very problematic)<br>①------②------③------④------⑤------⑥------⑦
① Physical difficulties (Pain, discomforts, etc.)	①------②------③------④------⑤------⑥------⑦
② Psychological difficulties (Anxiety, depression, stress, etc.)	①------②------③------④------⑤------⑥------⑦

Q6) How often did the pediatric patient **visit the hospital in the past six months?** Please write down the **approximate number of days** the patient visited **this hospital, another hospital, or a clinic** and categorize them according to the nature of the visit. **(All days in hospitalization should be included.)**

Categories	No. of days in the past six months
① Outpatient department (including laboratory follow up, physical therapy, etc.)	______________ days
② Admission (general ward)	______________ days
③ Admission (intensive care unit)	______________ days
④ Emergency department	______________ days
Total	______________ days

Q7) What **method of transportation** do you normally use to **travel from home to this hospital** with the pediatric patient? Please select **all** the transportation methods you use normally.

Copyright(c) 2019 Consensus Co. All rights reserved.

1. Private car
2. Intra-city bus / Subway
3. Train
4. Express bus / Intercity bus
5. Private ambulance
6. Public transportation support (Taxi for the disabled, etc.)
7. Other (Please specify;________________________________)

Q8) How long does it normally take for the pediatric patient to **travel from home to this hospital** for treatment?

1. Less than 30 min.
2. 30 min. ~ 1 hour
3. 1 ~ 2 hours
4. 2 ~ 3 hours
5. 3 ~ 4 hours
6. 4 ~ 5 hours
7. 5 ~ 6 hours
8. More than 6 hours

Q9) **How long do you wait** on average to receive **outpatient care** in this hospital? If the patient requires **outpatient services in two or more areas,** please select the **total waiting time**.

1. Less than 15 minutes
2. 15 ~ 29 minutes
3. 30 ~ 59 minutes
4. 60 ~ 89 minutes
5. 90 ~ 119 minutes
6. 120 ~ 179 minutes
7. More than 180 minutes

Q10) **How long** does **one outpatient appointment** with a doctor take on **average**?

1. Less than 5 minutes
2. 5 ~ 9 minutes
3. 10 ~ 14 minutes
4. 15 ~ 19 minutes
5. 20 ~29 minutes
6. more than 30 minutes

II. Questions on care of pediatric patients.

Q11) Who is currently **taking care of the pediatric patient at home**? Please write the number that represents **yourself or another individual who serves as the primary or secondary caregiver at home.**

Primary caregiver		Secondary caregiver	

1. Mother
2. Father
3. Grandmother
4. Grandfather
5. Expended families
6. Activity assistant/carer
7. Neighbor
8. Others (________________________________)

Q12) How does the **pediatric patient behave at home** on a regular basis?

1. Always lies on the bed or floor
2. Usually lies on the bed or floor, but sometimes sits or rides the wheelchair
3. Carries out some indoor activities with help from a guardian
4. Carries out indoor activities without major challenges

Q12-1) Please answer only if you selected 1 or 2 in [Question 12].
How often does the pediatric patient take or is the patient repositioned to the following positions while he or she is awake at home? Please select the closest answer for each category. This includes not only the patient

Copyright(c) 2019 Consensus Co. All rights reserved.

taking the position by himself or herself, but also the **guardian or caregiver repositioning the patient.**

Categories	Frequency of taking the position or being repositioned
① **Lying down**	1. Never 2. Seldom 3. Sometimes (more than once a week) 4. Often (more than once a day)
② **Lying down sideways** (left or right)	1. Never 2. Seldom 3. Sometimes (more than once a week) 4. Often (more than once a day)
③ **Sitting** (Includes using tools to help maintain posture)	1. Never 2. Seldom 3. Sometimes (more than once a week) 4. Often (more than once a day)
④ **Standing or walking** (Includes using standing aids)	1. Never 2. Seldom 3. Sometimes (more than once a week) 4. Often (more than once a day)

Q13) What does the pediatric patient do at home on a **typical day**? **Out of 24 hours in a day, how many hours does the patient spend on the following activities**

Categories	Average hours per day
① Sleep (day and night)	________________ hours/day
② Receiving medical care (taking medicine, suctioning, changing tracheostomy tube, etc.)	________________ hours/day
③ Receiving physical care (eating, bathing, dressing, etc.)	________________ hours/day
④ Play and rest (watching TV, surfing the internet, play games, reading, etc.)	________________ hours/day
⑤ Social activities (school, meeting friends, etc.)	________________ hours/day

Copyright(c) 2019 Consensus Co. All rights reserved.

Q14) On a typical day, what **medical treatment or practices** does the pediatric patient **receive at home**? **How often does the patient receive (such medical treatment or practices) in a day?**

Medical care		Average time or frequencies per day
① Home Mechanical Ventilator	1. Yes 2. No	________ hours/day
② Home Oxygen support	1. Yes 2. No	________ hours/day
③ Pulse Oximetry monitoring	1. Yes 2. No	________ hours/day
④ Nutrition support (levin tube, gastrostomy tube etc.)	1. Yes 2. No	________ times/day
⑤ Suction	1. Yes 2. No	________ times/day
⑥ Administering medications	1. Yes 2. No	________ times/day
⑦ Disinfection or cleaning of medical instrument/wound dressing	1. Yes 2. No	________ times/day
⑧ Rehabilitation therapy	1. Yes 2. No	________ times/day
⑨ Position change	1. Yes 2. No	________ times/day

Q15) What is the **most challenging or difficult aspect** of providing medical treatment or practices to the pediatric patient at home? Please select the **two** most challenging or difficult aspects in order and **write down their numbers**.

First		Second	

1. Assessing the pediatric patient's condition due to the lack of medical knowledge
2. Inexperience using equipment and providing medical practices
3. Making decisions during abnormal or emergency situations
4. Self-doubt or difficulty in confirming whether the correct care was provided
5. Providing the treatment alone without anyone's help
6. Show of discomfort or complaints of pain by the child
7. Other (Please specify ;________________________)

Q18) What do you do when you have a **medical question while taking care of the pediatric patient at home**? Please choose **two** methods that you normally use in order and **write down their numbers**.

First		Second	

1. Ask or search in web portals (Naver, Daum, etc.)
2. Ask or search in online patient associations or Internet forums
3. Ask a medical staff(doctor, nurse) via call or text
4. Ask another parent from a patient association or someone else via call or text
5. Look up professional literature or studies
6. Other (Please specify;__________)

Copyright(c) 2019 Consensus Co. All rights reserved.

Q19) Have you ever **asked a hospital staff member a medical question** via text, Kakao Talk, e-mail, etc. while taking care of the pediatric patient **at home in the past year**? Please select all the departments you have **asked at least once** in the past year.

1. Doctor in ward where child was previously hospitalized
2. Nurse working in the ward where the patient was hospitalized
3. Pediatric emergency department
4. Palliative care unit (Seoul National University Hospital)
5. Other (Please specify;____________________________________)

---

9. I have not asked any hospital staff in the past year

Copyright(c) 2019 Consensus Co. All rights reserved.

▣ Thee following questions are about **you, the respondent**.

Q24) What is the **most challenging or difficult aspect of taking care of the pediatric patient** for you today? Please select the **two** most challenging or difficult aspects in order and **write down their numbers**.

First		Second	

1. Inconveniences in daily life (sleep, meals, lack of private time, etc.)
2. Financial difficulties (medical expenses, etc.)
3. Anxiety about the condition of the pediatric patient
4. Lack of professional knowledge or information on illness and nursing
5. Negligence of family (Negligence in taking care of other children, family relations, etc.)
6. Lack of psychological stability and rest for the caregiver (parent)
7. Other (Please specify ;____________________________)

Q25) How does your day look like on a **typical day**? **Out of 24 hours in a day, how many hours do you spend on the following activities**?

Categories	Average hours per day
① Sleep	____________ hours/day
② Taking care of child (supervision, suctioning, feeding via tubes, positioning, etc.)	____________ hours/day
③ Household chores (vacuuming, laundry, taking care of other family members, etc.)	____________ hours/day
④ Private time and rest (watching TV, playing games, surfing the internet, reading, etc.)	____________ hours/day
⑤ Social activities (school, occupation, etc.)	____________ hours/day

Q26) Have you ever **reduced your workload or quit work** (job or other financial activities) to **take care of the pediatric patient**?

1. I have taken leave of absence, resigned, or temporarily stopped working
2. I am currently working but reduced my workload
3. I am continuing to work without any change
4. I am originally a homemaker

Q27) What is the **degree** of physical, psychological, and economic **difficulty** you feel while taking care of the pediatric patient at home? Please select between ‘A great deal (Very problematic)’(7) and ‘Not at all (Not a problem)’(1) for each difficulty.

Copyright(c) 2019 Consensus Co. All rights reserved.

Variables	Not at all (Not a problem) ①------②------③------④------⑤------⑥------⑦ A great deal (Very problematic)
① Physical difficulties (lack of sleep, fatigue, compromised physical health etc.)	①------②------③------④------⑤------⑥------⑦
② Psychological difficulties (Anxiety, depression, stress, etc.)	①------②------③------④------⑤------⑥------⑦
③ Economic difficulties (medical expenses, etc.)	①------②------③------④------⑤------⑥------⑦

Ⅲ. Questions on difficulties in taking care of the patient(s) at home.

Q30) Reflecting on your experience taking care of the pediatric patient at home, **how did you usually feel when faced with the following difficulties**? Please select between 'A great deal'(7) and 'Not at all'(1) for each category.

Variables	Not at all Somewhat A great deal ①------②------③------④------⑤------⑥------⑦
① I have experienced an embarrassment at home because I did not know about the pediatric patient' s condition or treatment method well	①------②------③------④------⑤------⑥------⑦
② I have experience failing to treat the pediatric patient because of inexperience with operating or managing equipment	①------②------③------④------⑤------⑥------⑦
③ I have experience not receiving help from the doctor or nurse for medical treatment	①------②------③------④------⑤------⑥------⑦
④ I have faced difficulties moving the pediatric patient when visiting the hospital	①------②------③------④------⑤------⑥------⑦
⑤ I have faced difficulties using hospital services (appointments, hospitalization/discharge, emergency room)	①------②------③------④------⑤------⑥------⑦
⑥ There is a medical treatment that I cannot administer alone (someone has to help)	①------②------③------④------⑤------⑥------⑦
⑦ I am always tired from taking care of the pediatric patient	①------②------③------④------⑤------⑥------⑦
⑧ I have been negligent in household chores due to the care for the pediatric patient	①------②------③------④------⑤------⑥------⑦
⑨ I have been negligent in caring for other children or family members from taking care of the pediatric patient	①------②------③------④------⑤------⑥------⑦
⑩ I have required someone' s help in caring for the pediatric patient	①------②------③------④------⑤------⑥------⑦
⑪ I have required someone' s help in finding stability and rest in myself and my family	①------②------③------④------⑤------⑥------⑦
⑫ I have had serious concerns regarding medical expenses for the pediatric patient	①------②------③------④------⑤------⑥------⑦
⑬ I have experience not providing required medical treatment or care due to financial burden	①------②------③------④------⑤------⑥------⑦

Q31) Did you ever feel that **medical treatment or practice was difficult or overwhelming** while taking care of a pediatric patient at **home**? **(If so,)** which medical treatment or practice is the **most difficult or overwhelming?**

1. Assessing the pediatric patient's condition
2. Administering medicine (oral administration, or needle injection)

Copyright(c) 2019 Consensus Co. All rights reserved.

3. Disinfecting/treating incisions or surgical sites
4. Using and managing equipment
5. Massaging, repositioning
6. Other (Please specify ;______________________)

---

9. None

Demographic Information

D 1) What is your gender?

1. Male 2. Female

D 2) What **age group** do you belong to?

1. 20s
2. 30s
3. 40s
4. 50s or older

D 3) Are you currently a **full-time homemaker**? Are you an **employed homemaker?**

1. Full-time homemaker
2. Employed homemaker (part-time job, freelance work, etc.)
3. Homemaker on leave of absence

---

9. Don't want to answer

D 4) What's the highest degree you earned?

1. Less than elementary school
2. Middle school
3. High school
4. Bachelor's degree
5. More than Graduate school

---

9. Don't want to answer

D 5) How does your **family structure** look like?

1. Parents + Underage child(ren)
2. Parents + Adult child(ren) + Underage child(ren)
3. Parents + Child(ren) + Grandparent(s)
4. Single parent + Child(ren)
5. Other (Please specify:)

---

9. Don't want to answer

D 6) How would you describe your **medical coverage**?

1. National Health Insurance
2. Medical Aid

---

9. Don't want to answer

D 7) Which of these categories best describes your family's level of economic status?

1. Upper
2. Upper-middle
3. Middle
4. Lower-middle
5. Lower

---

9. Don't know/Don't want to answer

D 8) Where is your current **region of residence**?

1. Seoul
2. Incheon / Gyeonggi
3. Busan / Ulsan / Gyeongnam
4. Daegue / Gyeongbuk
5. Daejeon / Sejong / Chungnam / Chungbuk
6. Gwangju / Jeonnam / Jeonbuk
7. Gangwon / Jeju

Copyright(c) 2019 Consensus Co. All rights reserved.
